# Supplementary material for: A Stereoselective Synthesis of a Novel α,β-Unsaturated Imine-Benzodiazepine through Condensation Reaction, Crystal Structure, and DFT Calculations
Source: Molecules. 2024 Sep 12;29(18):4323. doi: 10.3390/molecules29184323 (PMC11434389; doi:10.3390/molecules29184323)
Supplement: Supplementary file 1 [file molecules-29-04323-s001.zip › molecules-3172879-supplementary.pdf]

A Stereoselective Synthesis of a Novel α,β-Unsaturated Imine-Benzodiazepine through Condensation Reaction, Crystal Structure, and DFT Calculations

Samir Hmaimou[1], Marouane Ait Lahcen[1], Mohamed Adardour[1], Mohammed M.Alanazi[2], Atul Kabra[3], Mohamed Maatallah[1], and Abdesselam Baouid[1]

[1]Molecular Chemistry Laboratory, Department of Chemistry, Semlalia Faculty of Sciences, Cadi Ayyad University, Marrakech 40001, Morocco.
[2]Department of Pharmaceutical Chemistry, College of Pharmacy, King Saud University, Riyadh 11451, Saudi Arabia.
[3]University Institute of Pharma Sciences, Chandigarh University, Mohali 14041, Punjab, India.

Samir Hmaimou [1], Marouane Ait Lahcen [1], Mohamed Adardour [1], Mohammed M. Alanazi [2], Atul Kabra [3], Mohamed Maatallah [1,]* and Abdesselam Baouid [1]

1 Molecular Chemistry Laboratory, Department of Chemistry, Semlalia Faculty of Sciences, Cadi Ayyad University, Marrakech 40001, Morocco; samir.hmaimo@gmail.com (S.H.); mar.aitlahcen@gmail.com (M.A.L.); adardourmed@gmail.com (M.A.); baouid@uca.ac.ma (A.B.)
2 Department of Pharmaceutical Chemistry, College of Pharmacy, King Saud University, Riyadh 11451, Saudi Arabia; mmalanazi@ksu.edu.sa
3 University Institute of Pharma Sciences, Chandigarh University, Mohali 14041, Punjab, India; atul.e9963@cumail.in
* Correspondence: m.maatallah@uca.ma

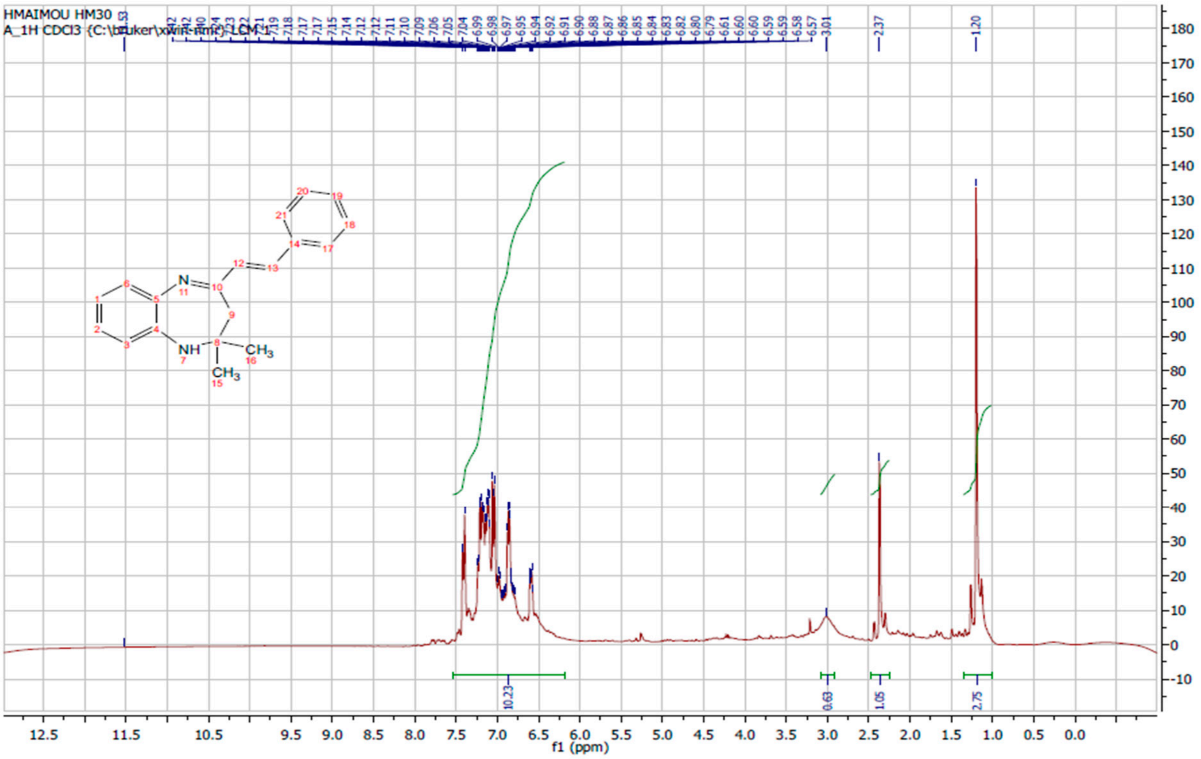

Figure S1: ^{1}H NMR spectrum of **3a** compound

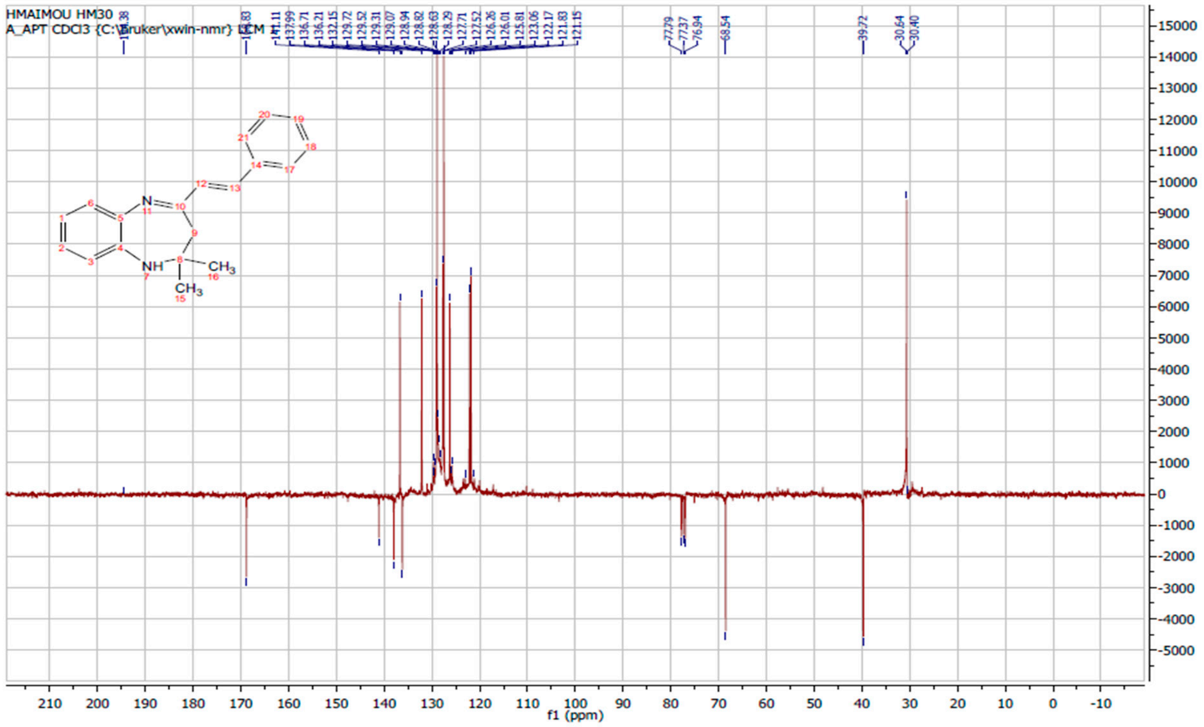

Figure S2: ^{13}C NMR spectrum of **3a** compound

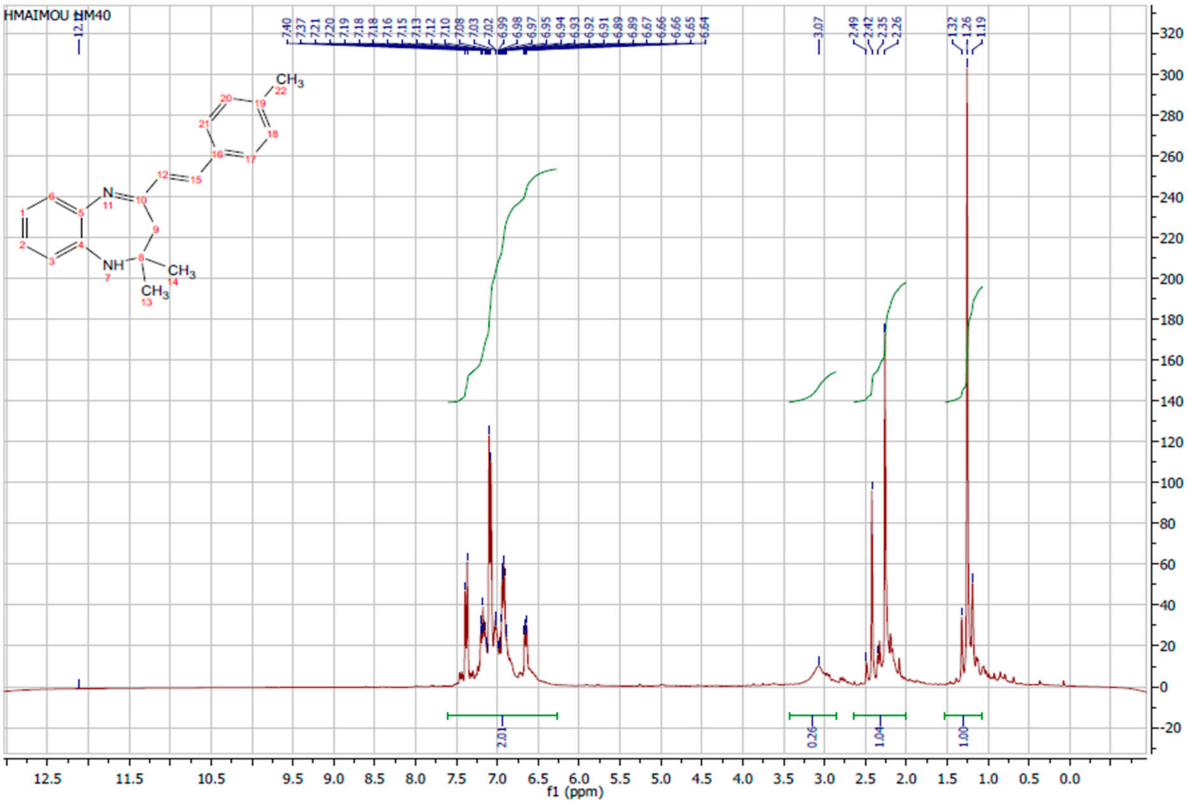

Figure S3: ^{1}H NMR spectrum of **3b** compound

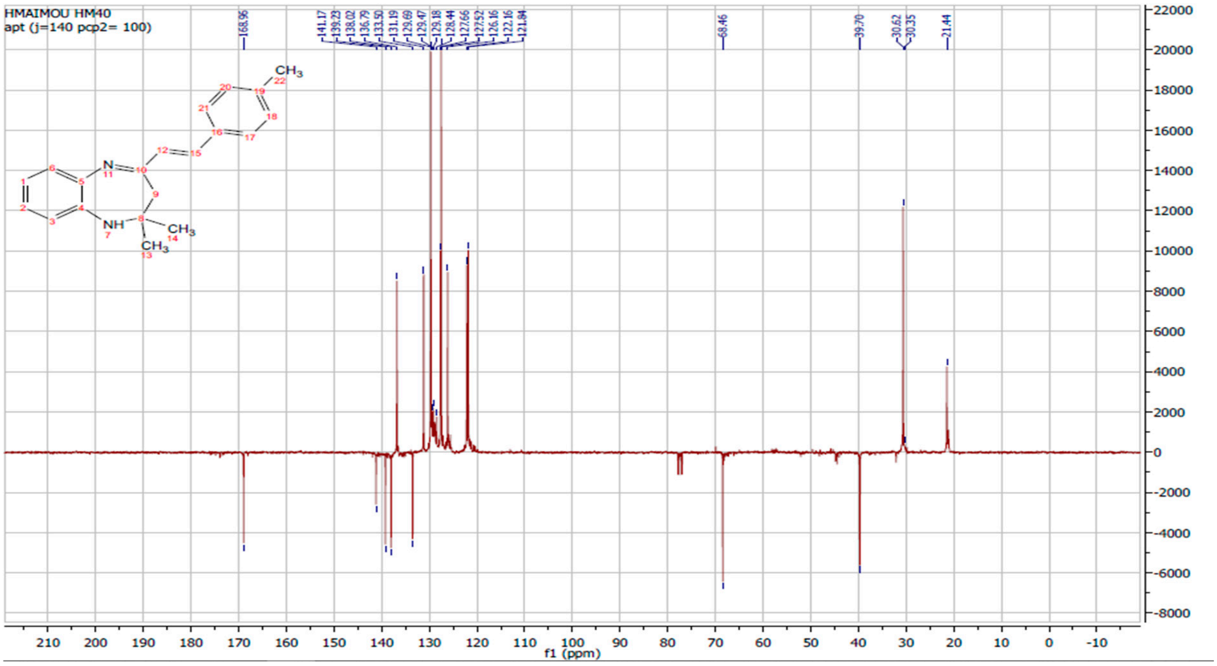

Figure S4: ^{13}C NMR spectrum of **3b** compound

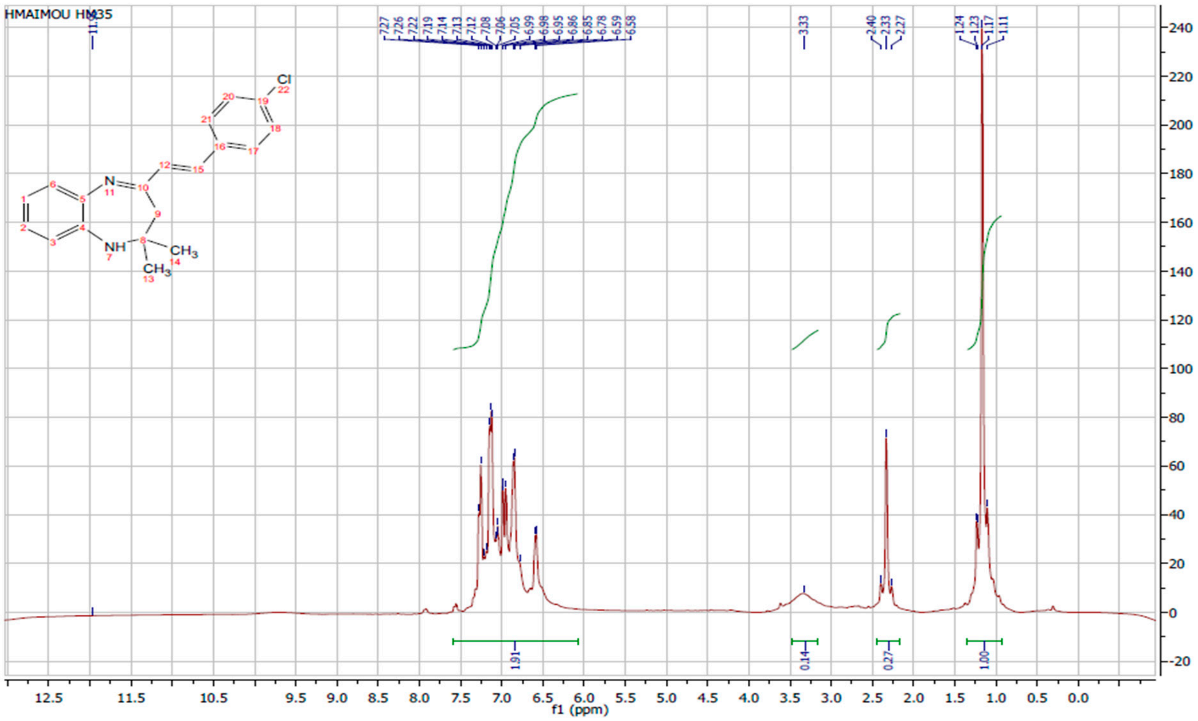

Figure S5: ^{1}H NMR spectrum of **3c** compound

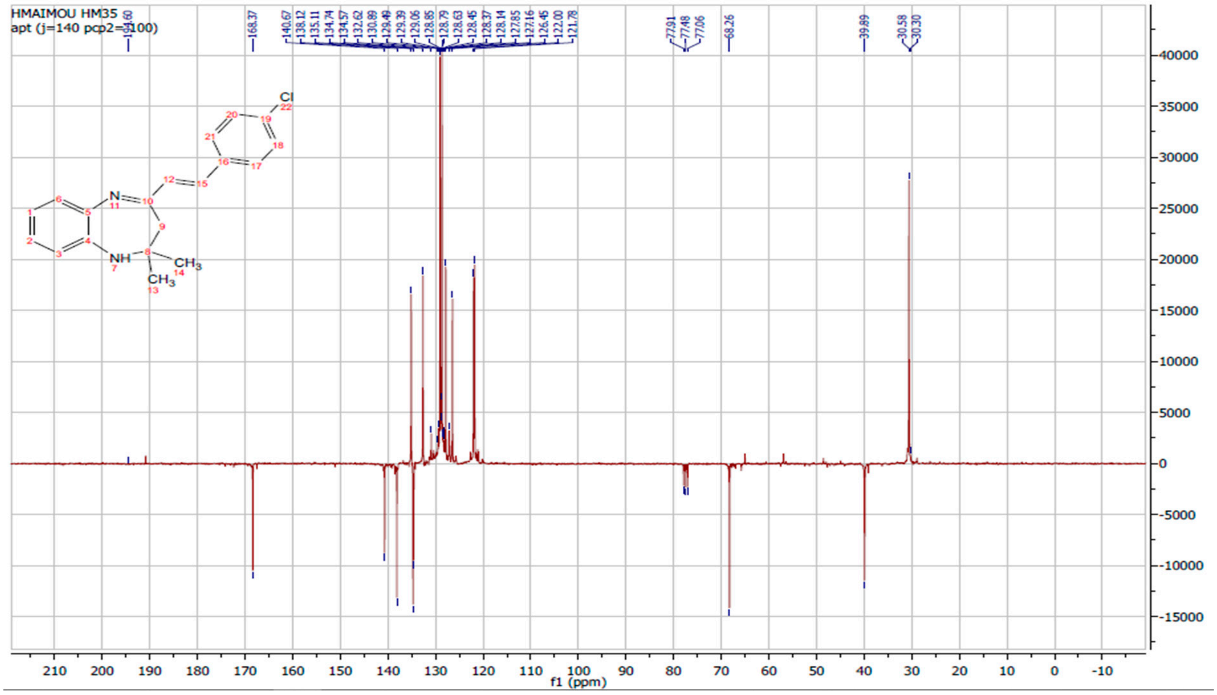

Figure S6: ^{13}C NMR spectrum of **3c** compound

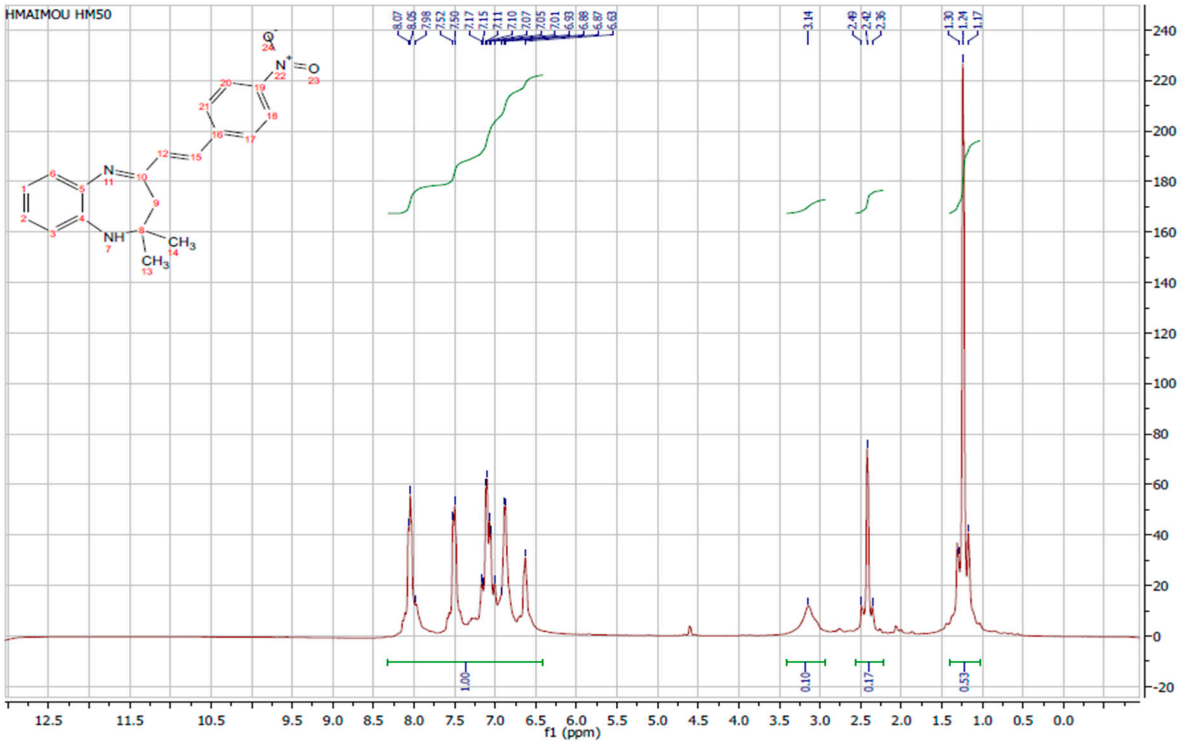

Figure S7: ^{1}H NMR spectrum of **3d** compound

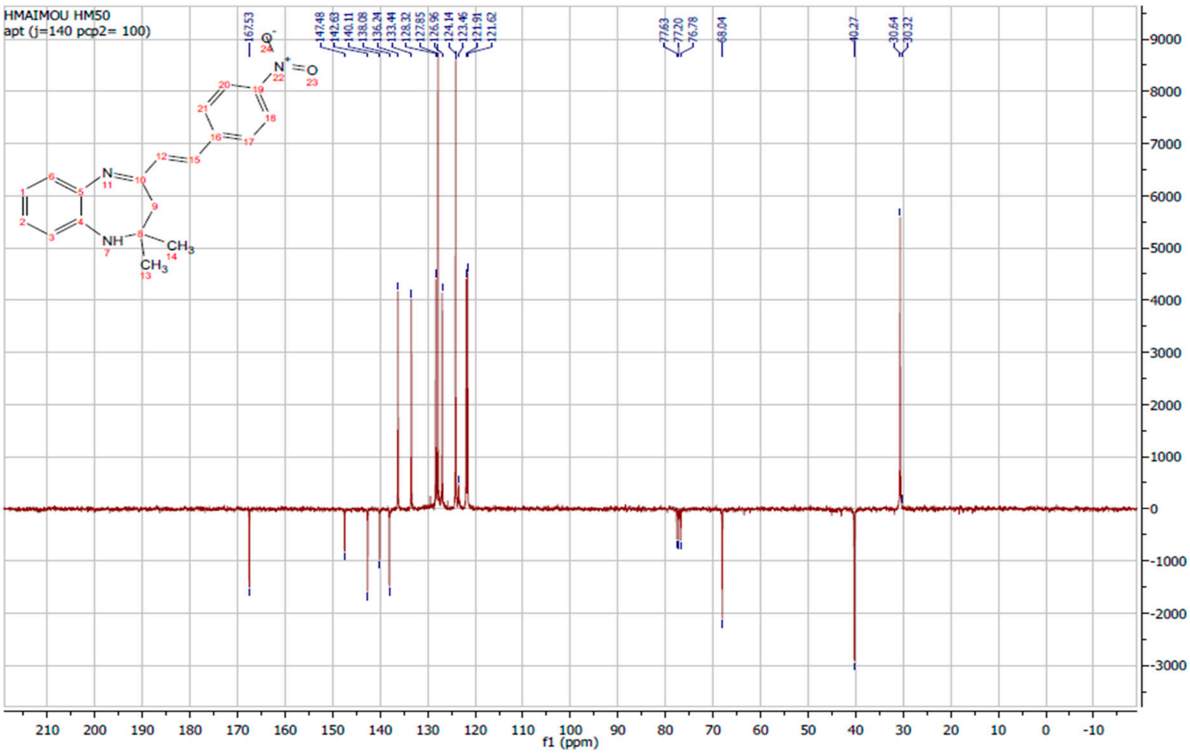

Figure S8: ^{13}C NMR spectrum of **3d** compound

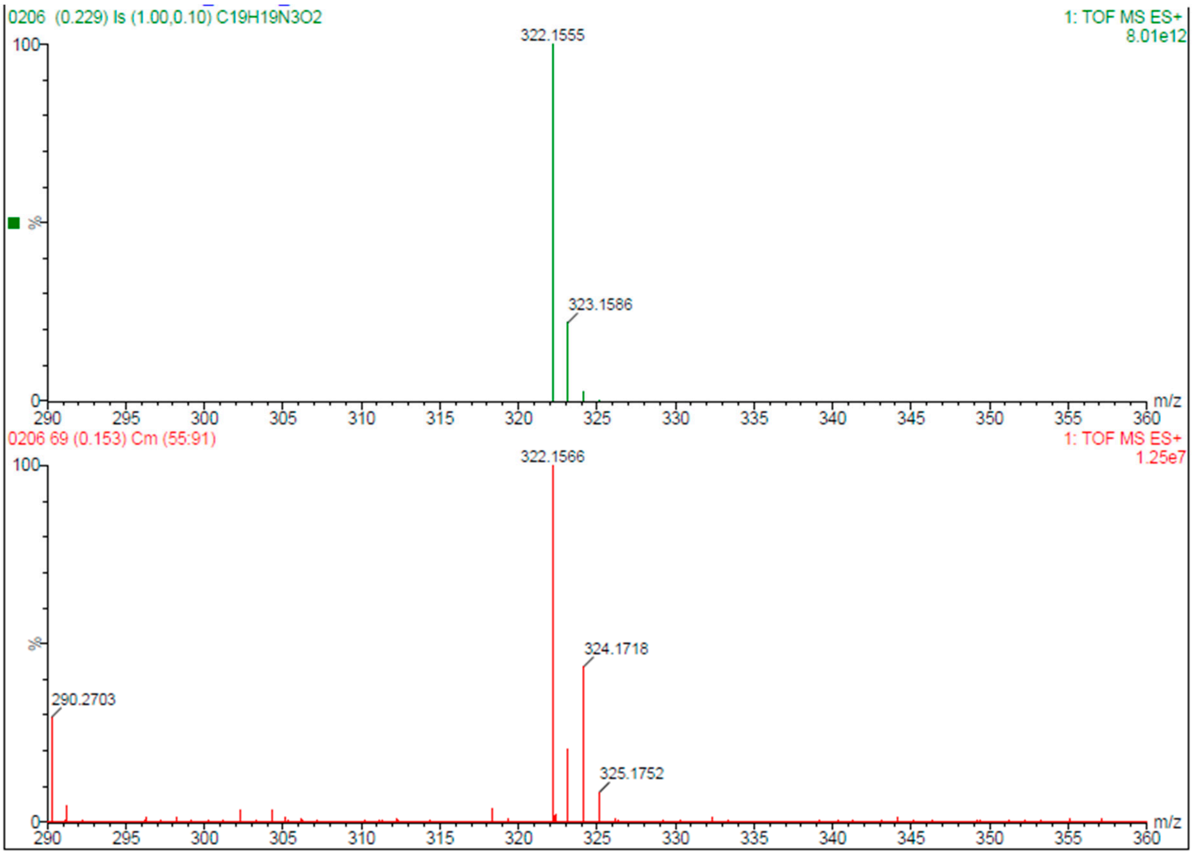

Figure S9: HRMS spectrum of **3d** compound
